# Artificial-Intelligence-Based Cephalometric Landmark Detection in Lateral Cephalograms

**DOI:** 10.3390/jcm15155943

**Published:** 2026-07-30

**Authors:** Manami Yamaguchi, Masato Tsutsumi, Yasuhiro Kuroda, Yoshiki Soeda, Tetsutaro Yamaguchi

**Affiliations:** 1 Department of Orthodontics, School of Dentistry, Kanagawa Dental University, Yokosuka 238-8580, Japan; t.yamaguchi@kdu.ac.jp; 2 Life Science Center for Survival Dynamics, Tsukuba Advanced Research Alliance, 1-1-1 Tennodai, Tsukuba 305-8577, Japan; mtsutsumi@tara.tsukuba.ac.jp; 3 Laboratory of Data-Driven Biology, Department of Integrative Cellular Informatics, Center for Neurological Diseases and Cancer, Nagoya University Graduate School of Medicine, Tsurumai 65, Showa-ku, Nagoya 466-8550, Japan; 4 EDIAND Inc., Tokyo 135-0062, Japan; kuroda@ediand.co.jp (Y.K.); soeda@ediand.co.jp (Y.S.)

**Keywords:** Artificial Intelligence, lateral cephalogram, diagnostic imaging, deep learning

## Abstract

**Background/Objectives:** Accurate landmark identification underpins reliable cephalometric analysis. This study evaluated a ResNet50-based, single-stage regression convolutional neural network for direct automatic localization of 15 landmarks on lateral cephalograms. **Methods**: This retrospective study included 669 lateral cephalograms (669 patients): 619 for training and 50 randomly selected for an internal holdout test set. One of five orthodontists annotated each cephalogram, and coordinates served as the reference standard. One orthodontist independently re-annotated all test images after more than 2 weeks to assess reproducibility. Model performance was evaluated using Euclidean localization errors and success detection rates (SDR). **Results**: Across 750 landmark predictions, mean localization error was 1.25 ± 1.39 mm (95% confidence interval, 1.15–1.35 mm) and median error was 0.72 mm. SDRs within 1.0, 2.0, and 4.0 mm were 64.5%, 81.5%, and 94.4%, respectively. A statistically significant overall difference was observed among the 15 landmarks (Friedman χ^2^(14) = 28.84, *p* = 0.011). Point A had the numerically largest mean error (1.66 mm) and the mandibular central incisor the smallest (1.04 mm). The mean difference between original and repeated annotations was 0.52 ± 0.92 mm. **Conclusions**: In this single-center internal holdout test set, mean localization error was below the 2.0 mm benchmark. However, 18.5% of predictions exceeded 2.0 mm, and external validity remains unconfirmed. Artificial-intelligence-generated landmarks should be verified by orthodontists, and external validation is required before broader clinical use.

## 1. Introduction

In orthodontic diagnosis and treatment planning, lateral cephalometric analysis remains a fundamental, standardized method for quantitative assessment [1,2,3,4]. However, conventional analysis relies on manual landmark identification and measurement, raising concerns about the time required for analysis [5,6,7], interexaminer variability, and reproducibility [8]. Landmark identification also depends heavily on examiner experience and skill; discrepancies have been reported both among examiners and across repeated measurements by the same examiner [8]. Therefore, specialized training and repeated practice are required to perform cephalometric analysis accurately and consistently [8].

In recent years, Artificial Intelligence (AI) has progressed rapidly in healthcare [9,10], with substantial performance gains reported, particularly in medical image analysis [9,10,11].

In orthodontics, numerous studies have applied AI to lateral cephalograms [12,13,14]. These AI-based approaches have attracted attention as tools for evaluating maxillofacial morphology and supporting treatment planning [12,15,16,17,18,19,20].

Because AI model performance in diagnosis and treatment planning depends on accurate, reproducible cephalometric inputs, errors in landmark identification may influence downstream diagnostic outcomes [21,22]. Automatic landmark identification has been investigated in both two-dimensional and three-dimensional cephalometric imaging [23]. Although cone-beam computed tomography enables three-dimensional evaluation of maxillofacial structures, its routine clinical use remains limited because of concerns about radiation exposure [24]. Therefore, lateral cephalograms continue to be widely used in orthodontic diagnosis and treatment planning [4].

Prior studies have applied different network architectures for cephalometric landmark detection, including single-stage object-detection methods such as YOLOv3 and SSD [25] as well as two-stage convolutional neural network (CNN) pipelines [26]. Broader reviews have summarized AI and CNN applications in dentistry [27,28], and fully automated quantitative cephalometry has also been implemented using CNNs [29]. In sequential or cascaded frameworks, errors introduced during initial localization may propagate to subsequent refinement steps [6,26]. Nonetheless, whether a single-stage coordinate-regression approach provides any practical or performance advantage still requires direct comparative evaluation.

Therefore, this study evaluated the localization accuracy of a ResNet50-based single-stage regression convolutional neural network for directly estimating 15 hard-tissue landmarks, using a single-center internal holdout test set.

## 2. Materials and Methods

### 2.1. Data Collection

This retrospective cross-sectional study analyzed 669 lateral cephalograms acquired during routine orthodontic care at the Department of Orthodontics, Kanagawa Dental University Hospital, between 1 April 2019 and 31 December 2024. The radiographs were obtained for clinical purposes before the initiation of this study and were not acquired specifically for research. Lateral cephalograms were acquired during routine orthodontic care using a cephalometric X-ray diagnostic unit (model CX-150W; Asahi Roentgen Ind. Co., Ltd., Kyoto, Japan) equipped with an X-ray tube manufactured by Shimadzu Corporation (Kyoto, Japan). Because of the retrospective design, imaging parameters were not prospectively standardized for this study. Therefore, the dataset reflected routine clinical variability in patient positioning, exposure conditions, and image contrast within a single-center clinical workflow. Patients with congenital disorders or systemic diseases that could affect craniofacial growth or morphology were excluded to reduce heterogeneity related to conditions outside the target population of this initial model-development study. Patients with mandibular deviation were also excluded because marked asymmetry may increase bilateral structural superimposition on lateral cephalograms and affect landmark identification. Mandibular deviation was defined as displacement of Menton greater than 2 mm from the facial midline.

This retrospective study, involving secondary use of existing clinical radiographs, was conducted in accordance with the Declaration of Helsinki and was approved by the Kanagawa Dental University Research Ethics Review Committee on 26 January 2026 (Approval No. 1143). No additional radiographs were acquired for this study. As only anonymized existing clinical data were used, the requirement for written informed consent was waived by the Ethics Committee. Information about the study was disclosed via an opt-out notice, and patients were given the opportunity to decline use of their data.

**Figure 1:**
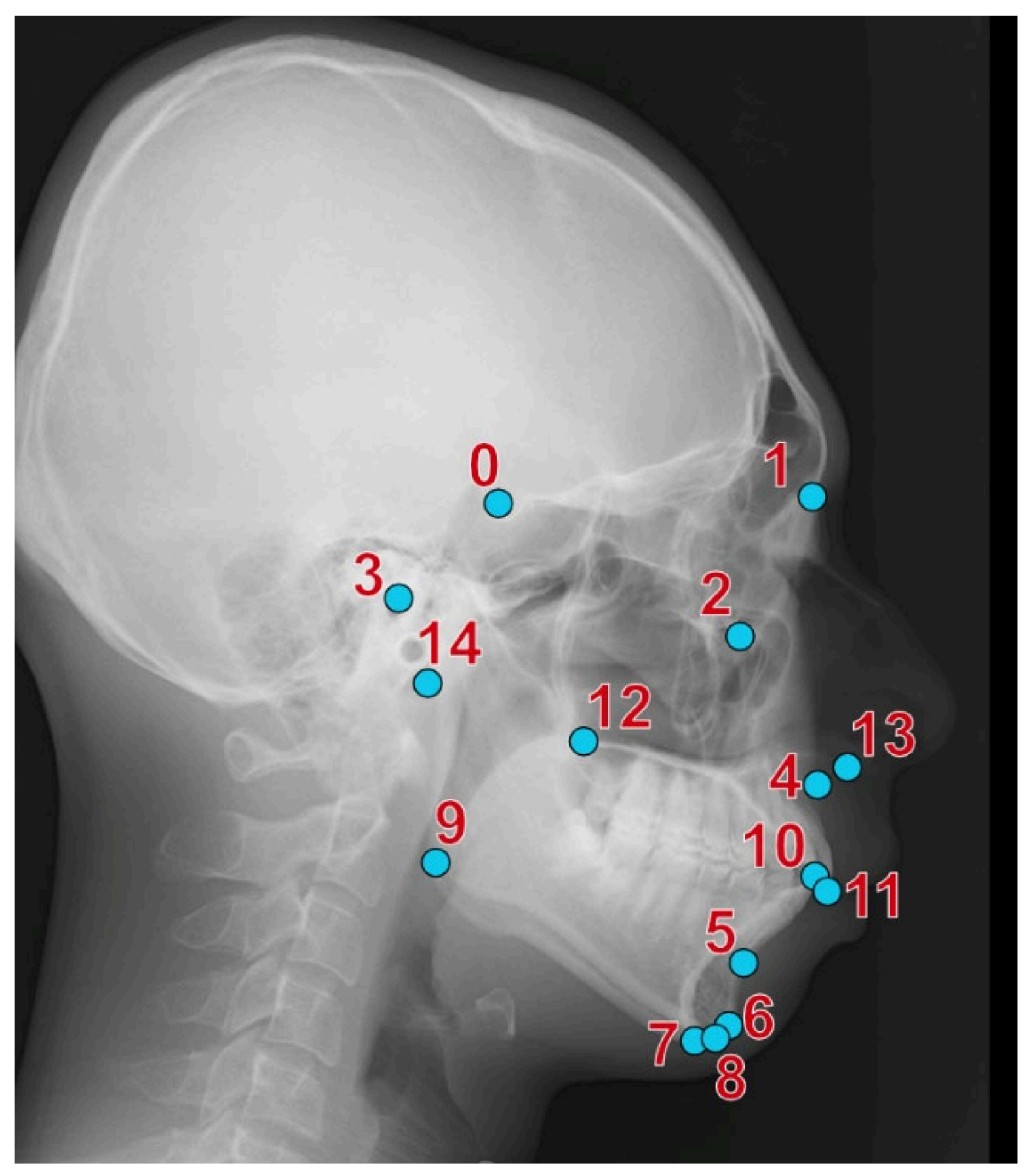
Cephalometric landmarks. Fifteen landmarks were defined on the lateral cephalogram as follows: (0) Sella; (1) Nasion; (2) Orbitale; (3) Porion; (4) Point A; (5) Point B; (6) Pogonion; (7) Menton; (8) Gnathion; (9) Gonion; (10) md1-Incisor inferius; (11) mx1-Incisor superius; (12) Posterior nasal spine; (13) Anterior nasal spine; and (14) Articulare.

CephaloMetrics AtoZ software (version 20.00; YASUNAGA Computer Systems Co., Inc., Fukui, Japan) was used for landmark measurement. Each image was resized to 720 × 580 pixels, and 15 anatomical landmarks were then identified on each cephalogram according to predefined anatomical definitions (Figure 1; Table 1).

Each of the 669 lateral cephalograms was assigned to one of five orthodontists. For each image, all 15 anatomical landmarks were manually identified by the assigned orthodontist, and the *x*- and *y*-coordinates were recorded according to the predefined anatomical definitions. Thus, each cephalogram was annotated by a single orthodontist. The coordinates were not averaged across the five orthodontists. The coordinates recorded for each image were used as the reference-standard coordinates. This reference-standard annotation procedure was applied to both the training and test datasets.

To assess the reproducibility of the reference landmark annotations, one of the five orthodontists independently re-annotated the 15 landmarks on all 50 test-set cephalograms after an interval of more than two weeks. Each test image had originally been annotated by one of the five orthodontists rather than by all five orthodontists. Therefore, this assessment evaluated agreement between the original clinical annotation and an independent remeasurement performed by a single examiner. As the original annotations were performed by different orthodontists, whereas all repeated annotations were performed by a single orthodontist, this analysis represented a mixed reference–annotation reproducibility assessment rather than a fully crossed interexaminer reliability study. Therefore, it did not quantify true interexaminer variability among all five orthodontists.

Among the 669 eligible patients, 619 were allocated to the training set and 50 to the internal holdout test set. Only one lateral cephalogram per patient was included in the dataset, and the 50 test cases were randomly selected before model training. Because each patient contributed a single image, there was no patient overlap between the training and test sets, thereby preventing patient-level data leakage. Age, sex, skeletal pattern, and image quality were not used as stratification factors during test-set selection. The test set comprised 20 male and 30 female patients. The mean ages of male and female patients were 18.2 years (range, 7.3–45.3 years) and 27.2 years (range, 7.5–65.2 years), respectively. The internal holdout test set was used to assess model performance on data not used for training.

### 2.2. Image Preprocessing

Standard lateral cephalometric radiographs in Digital Imaging and Communications in Medicine format were loaded, and pixel values were normalized by dividing each pixel value by the maximum pixel value. Normalized images were resized to 720 × 580 pixels (height × width) before model training and testing, using the same preprocessing procedure for all images. This preprocessing was applied to reduce variability in image size and intensity scaling. Nonetheless, it does not fully account for differences that may arise from different radiographic systems, sensors, exposure protocols, or institutional imaging workflows. To match the deep-learning model’s input format, grayscale images were converted to three-channel images.

Data augmentation was applied to the training data.

Augmentation techniques included brightness and contrast adjustments, image downsampling, translation, scaling, and rotation. Brightness and contrast adjustments were applied with a probability of 20%. Image downsampling was applied with a probability of 50%, using a scale range of 0.75–0.99. Geometric transformations comprised translations within ±10% of the image width and height, scaling of 0–30%, and rotations within ±5°. During geometric transformations, the corresponding landmark coordinates were transformed in the same manner as the images. Data augmentation was applied only to the training data.

### 2.3. Deep-Learning Model Architecture

A deep-learning model was constructed using ResNet50 pretrained on ImageNet as the base model and adapted through transfer learning to estimate landmark coordinates on standard lateral cephalometric radiographs. ResNet50 is a convolutional neural network with residual connections that extracts image features while mitigating vanishing gradients in deep architectures.

To address the limited dataset of 669 images, transfer learning was performed using a ResNet50 model pre-trained on ImageNet. As the initial layers of the pretrained model extract general image features, including edges, brightness gradients, and local textures, they were considered useful for extracting morphological features, such as bone contours and teeth, from lateral cephalometric radiographs. However, as natural images and lateral cephalometric radiographs represent different image domains, only the first 82 layers were frozen, whereas the subsequent layers and newly added fully connected layers were trained for landmark-coordinate estimation.

**Figure 2:**
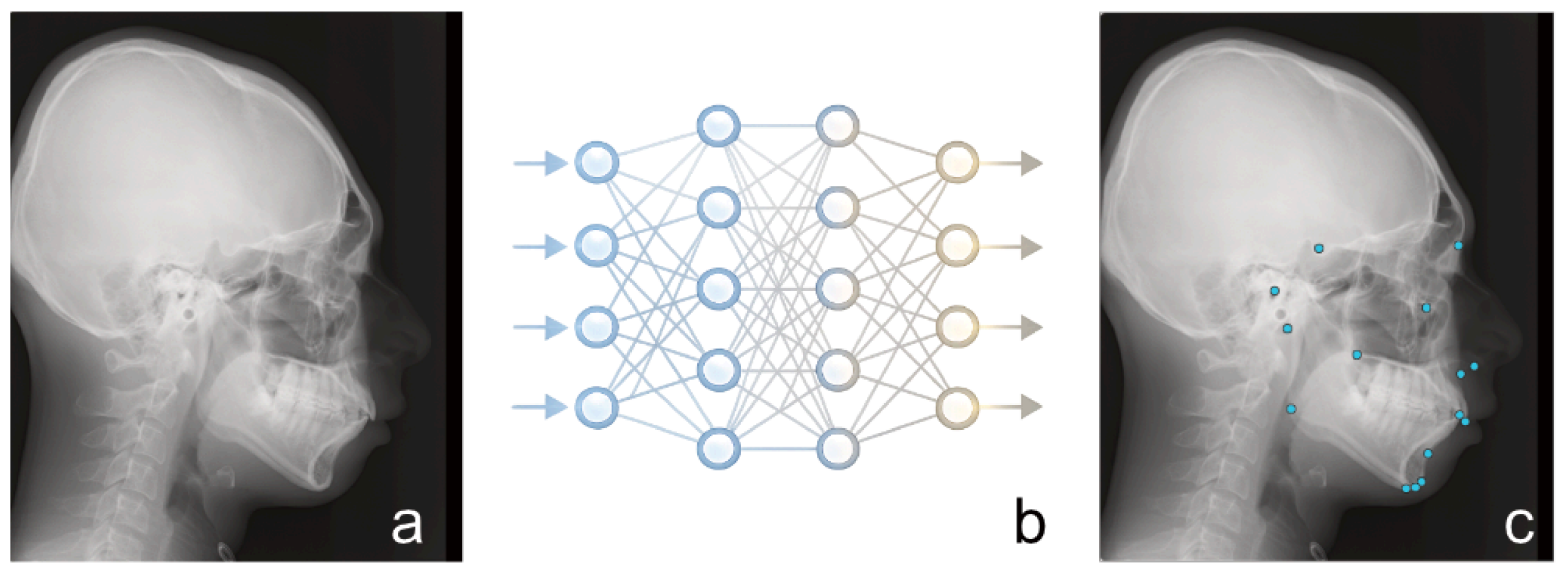
Deep-learning model workflow. (**a**) Input lateral cephalogram; (**b**) convolutional neural network-based feature extraction and landmark-coordinate regression; and (**c**) predicted landmark coordinates.

The ResNet50 classification layer was removed, and a single-stage regression-based convolutional neural network (CNN) was constructed to directly estimate 15 landmark coordinates from standard lateral cephalometric radiographs (Figure 2). The model input size was 720 × 580 × 3. A 4 × 4 max-pooling layer was connected to the ResNet50 output feature maps, which were then converted into one-dimensional vectors using a Flatten layer. Fully connected layers with 256 and 128 units were then added sequentially, each using the rectified linear unit as the activation function. The final fully connected layer contained 30 units, outputting the *x*- and *y*-coordinates of the 15 landmarks as continuous values. No activation function was used in the output layer.

### 2.4. Model Training

The model was trained using the Huber loss function (delta = 0.1). Optimization was performed with stochastic gradient descent. The learning rate, momentum, clip norm, and batch size were set to 0.1, 0.9, 0.5, and 32, respectively. The clip norm was used to constrain excessively large gradient updates.

During training, the training data were shuffled and used for mini-batch training. Data-augmented images, together with the corresponding reference-standard landmark coordinates, were used as inputs. Model parameters were updated to minimize the error between the model’s 30-dimensional vector output and the coordinates manually recorded by the orthodontist assigned to each image. A validation set was used to monitor the Huber loss during training, and training was conducted for up to 5000 epochs. If the validation loss did not improve for 90 consecutive epochs, the learning rate was reduced by a factor of 0.1. Early stopping was applied when the validation loss remained unchanged for 200–400 epochs. The model weights yielding the lowest validation loss observed across the entire training process were retained for final evaluation.

**Figure 3:**
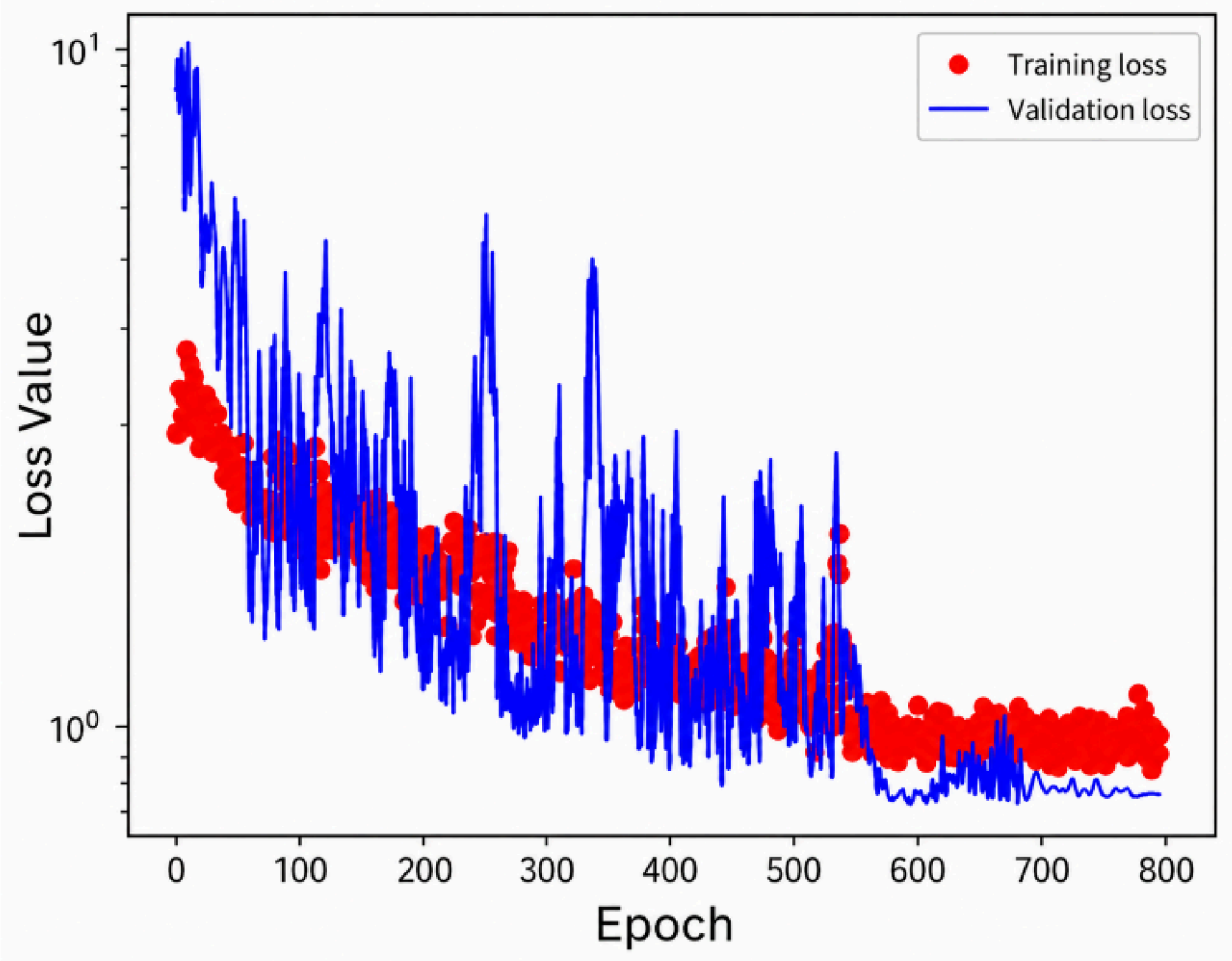
Training and validation loss curves over the course of model training. The *y*-axis presents the loss value on a logarithmic scale, whereas the *x*-axis indicates the number of training epochs. Red dots represent the training loss, and the blue line represents the validation loss.

Hyperparameters, including a batch size of 32, were chosen empirically. Data augmentation, as described in Section 2.2, was applied to reduce overfitting given the relatively small dataset. The training and validation loss curves were monitored throughout model development (Figure 3). Although the validation loss fluctuated during the early and middle stages of training, both losses decreased over time and subsequently stabilized. Repeated training with different random seeds was not performed; therefore, variability across independent training runs was not assessed.

### 2.5. Evaluation Metrics

**Figure 4:**
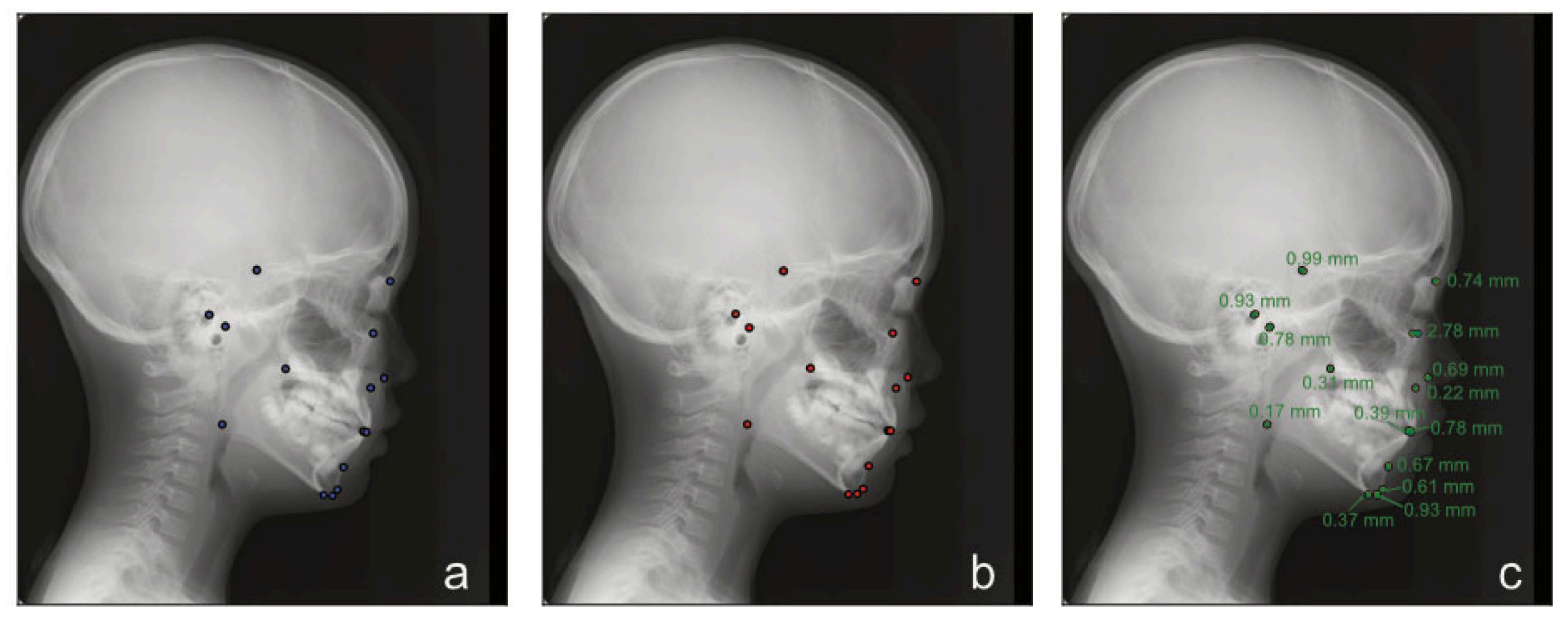
Definition of measurement error. (**a**) Manual reference-standard landmarks (blue dots). (**b**) AI-predicted landmarks (red dots). (**c**) Euclidean distances between corresponding manual and AI-predicted landmarks.

Model performance was evaluated by calculating, for each landmark, the Euclidean distance between the reference-standard coordinates manually recorded on each test image and the coordinates predicted by the AI model. Coordinate error was defined as this Euclidean distance, computed from the squared differences in the *x*- and *y*-coordinates (Figure 4).

With the reference-standard coordinates of landmark i denoted as ($x_{i}$, $y_{i}$) and the model-predicted coordinates denoted as (${\overset{‵}{x}}_{i},{\overset{‵}{y}}_{i}$), the error was calculated using the following equation:

$${error}_{i}\;=\;\sqrt{{\left(x_{i}-{\hat{x}}_{i}\right)}^{2}+{\left(y_{i}-{\hat{y}}_{i}\right)}^{2}}$$

For each landmark, the error was calculated across all test images, and the mean error, standard deviation, and median were then computed.

Localization errors were calculated in the coordinate system of the resized 720 × 580-pixel model-input images and subsequently converted to millimeters using a case-specific conversion factor. The DICOM Pixel Spacing attribute was not used. Instead, the conversion factor was estimated by aligning the metric coordinate system exported from CephaloMetrics AtoZ with the corresponding resized DICOM image. In practical terms, this procedure determined how many pixels in each resized image corresponded to 1 mm in the AtoZ coordinate system.

First, the AtoZ landmark coordinates were aligned with the tracing image using the Sella–Nasion reference line, with Sella defined as the origin. The tracing image was then registered to the corresponding DICOM image using AKAZE feature matching, Lowe’s ratio test with a threshold of 0.7, and RANSAC-based estimation of a partial affine transformation. By combining these transformations, a mapping was obtained from the AtoZ coordinate system, expressed in millimeters, to the resized image coordinate system, expressed in pixels.

The case-specific pixel-to-millimeter conversion factor was derived from the scaling component of this combined transformation. Let A denote the 2×2 linear component of the transformation. The effective scale was calculated as $\sqrt{|\det\left(A\right)|\;}$ pixel/mm, and its reciprocal, $1/\sqrt{|det\left(A\right)|}\;$ mm/pixel, was used to convert pixel-based Euclidean localization errors into millimeters. Translation was not included in the scale calculation because it changes the image’s position but not its distance.

To identify obvious registration or conversion errors, the transformed landmark positions were overlaid on the corresponding DICOM images and visually inspected for misalignment. No obvious registration failures were identified during this inspection. However, the accuracy of the conversion factor was not formally validated using an independent quantitative reference, such as the DICOM Pixel Spacing attribute or an external calibration phantom. Therefore, residual uncertainty in the millimeter conversion should be considered a methodological limitation of this study.

### 2.6. Statistical Analysis

Statistical analyses were performed using IBM SPSS Statistics, version 26.0 (IBM Corp., Armonk, NY, USA). The normality of the prediction-error distributions was assessed using the Shapiro–Wilk test. As the error distributions were not normally distributed, nonparametric statistical tests were applied.

For patient-level analyses, the mean error across the 15 landmarks was calculated for each of the 50 patients. These patient-level mean errors were then compared between female and male patients and between patients younger than 18 years and those aged 18 years or older using the Mann–Whitney U test. The 18-year cutoff was used pragmatically to distinguish younger patients from adults and was not intended to represent skeletal maturity.

For landmark-level analyses, prediction errors for each landmark across the 50 test cases were evaluated. Differences in prediction error across the 15 landmarks were assessed using the Friedman test. Because the overall Friedman test result was statistically significant, post hoc pairwise comparisons between individual landmarks were performed using two-sided Wilcoxon signed-rank tests with Holm adjustment for multiple comparisons.

The overall mean error and its 95% confidence interval were calculated using all 750 landmark errors from the 50 test cases and 15 landmarks. The confidence interval was derived from the sample mean and standard deviation using the corresponding t distribution. All statistical tests were two-sided, and *p* values below 0.05 were considered statistically significant. For the reference–annotation reproducibility analysis, the original and repeated landmark coordinates were expressed in a common coordinate system before analysis. Agreement between the original and repeated annotations performed by the same examiner was summarized separately for the *x*- and *y*-coordinates using a two-way mixed-effects, absolute-agreement, single-measurement intraclass correlation coefficient. Intraclass correlation coefficient (ICC) values were calculated for each landmark. As the same test images were not independently annotated by all five orthodontists, these ICC values should not be interpreted as estimates of interexaminer reliability among all five orthodontists. In addition, the Euclidean distance between the original and repeated coordinates was calculated for each landmark, and the mean distance and the proportion of paired annotations within 2.0 mm were determined.

## 3. Results

### 3.1. Overall Error Trend

In the 50 test cases, Euclidean distances were calculated between the deep-learning model-predicted landmark coordinates and the corresponding reference-standard coordinates manually recorded for each image. Across all landmarks (750 points), the mean error was 1.25 ± 1.39 mm (95% confidence interval, 1.15–1.35 mm), and the median error was 0.72 mm.

### 3.2. Reference–Annotation Reproducibility

The mean Euclidean difference between the original and repeated landmark annotations was 0.52 ± 0.92 mm, and 93.3% of paired annotations fell within 2.0 mm. Excluding Nasion, for which the ICC could not be calculated because it was used as the coordinate origin, landmark-specific ICCs ranged from 0.982 to 1.000 for the *x*-coordinates and from 0.765 to 1.000 for the *y*-coordinates. Porion had the largest mean examiner-related difference (2.81 ± 1.70 mm) and the lowest ICC for the *y*-coordinate (ICC = 0.765), whereas the other landmarks generally demonstrated smaller differences and higher agreement. These results describe agreement between the original annotations and the repeated annotations performed by the same examiner and do not represent a fully crossed assessment of interexaminer reliability among the five orthodontists.

### 3.3. Patient-Level Error Distribution

**Figure 5:**
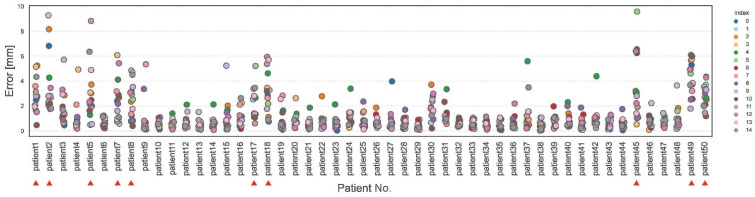
Distribution of landmark errors by patients. Colored dots denote the localization errors for the 15 landmarks in each patient. Red triangles beneath the *x*-axis mark cases identified as statistical outliers.

Figure 5 shows the error distribution across the 15 landmarks for each patient. Outlier analysis using the Q3 + 1.5 × IQR criterion identified 10 of the 50 cases (No. 1, No. 2, No. 5, No. 7, No. 8, No. 17, No. 18, No. 45, No. 49, and No. 50) as outliers; their mean errors exceeded the 2.15 mm threshold. Among these, No. 2, a 26-year-old male patient with a mean error of 3.63 mm, showed marked maxillary protrusion, with a Sella-Nasion-A point (SNA) angle of 89.8° and an ANB angle of + 9.4°. No. 49, a 16-year-old female patient with a mean error of 4.31 mm, showed an extremely hypodivergent skeletal pattern, with a Frankfort mandibular plane angle (FMA) of 5.8°.

A descriptive review of all 10 statistical outlier cases did not identify a consistent pattern attributable to a single landmark or skeletal morphology. Some cases showed uncommon craniofacial characteristics, whereas others had relatively large errors at landmarks in regions of low image contrast, along curved anatomical contours, or in areas with bilateral structural overlap. Because this analysis was exploratory, these observations should not be interpreted as evidence of an association between specific craniofacial characteristics and prediction error.

Patient characteristics associated with measurement error were also examined (Figure 5). Patient-level mean errors did not differ significantly by sex, with values of 1.24 ± 1.03 mm in female patients (*n* = 30) and 1.25 ± 0.91 mm in male patients (*n* = 20, Mann–Whitney’s U test, *p* = 0.641). Similarly, no significant difference was observed between patients younger than 18 years (*n* = 19; mean error, 1.35 ± 1.12 mm) and those aged 18 years or older (*n* = 31, mean error, 1.19 ± 0.89 mm, *p* = 0.867). No statistically significant differences were observed by sex or age group. Nonetheless, because these subgroup analyses were exploratory and based on a relatively small test set, the findings should not be interpreted as evidence of equivalent model performance across patient characteristics.

### 3.4. Landmark-Specific Error Distribution

**Figure 6:**
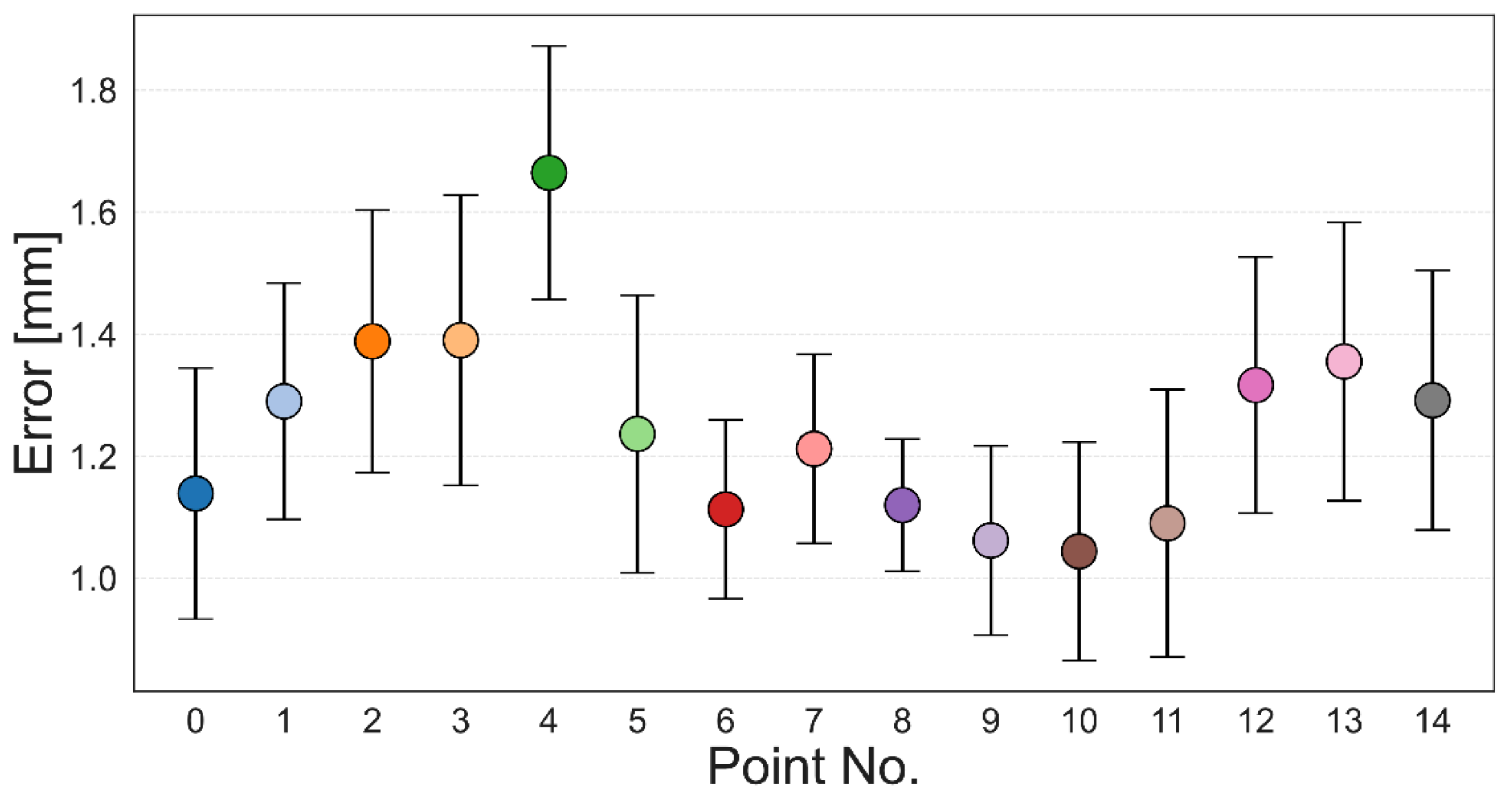
Landmark-specific localization errors. Points denote the mean localization error for each landmark, and error bars represent the standard error of the mean. Landmark-specific means, standard deviations, and success detection rates are provided in Table 2.

The error distribution for each landmark is shown in Figure 6. Landmark-level prediction errors were compared among the 15 landmarks using the Friedman test. A statistically significant overall difference was detected among the landmarks (χ^2^(14) = 28.84, *p* = 0.011). Accordingly, post hoc pairwise comparisons were performed using two-sided Wilcoxon signed-rank tests with Holm adjustment for multiple comparisons. The prediction error for Point A was significantly greater than that for md1-Incisor inferius (adjusted *p* = 0.035), whereas no other pairwise comparison remained statistically significant after Holm adjustment. Descriptively, landmarks in regions of bilateral overlap or relatively low image contrast, including Orbitale, Porion, Point A, PNS, and ANS, had a mean error of 1.42 mm. Landmarks in relatively high-contrast regions, including Gnathion, Gonion, md1-Incisor inferius, and mx1-Incisor superius, had a mean error of 1.08 mm. These group values are descriptive and were not subjected to confirmatory statistical comparison. Among the individual landmarks, Point A showed the numerically largest mean error (1.66 mm), whereas md1-Incisor inferius showed the numerically smallest mean error (1.04 mm).

**Table 2:** Prediction errors and success detection rates by landmarks.

Landmark	Mean (mm)	SD	SDR ≤ 1.0 mm	SDR ≤ 2.0 mm	SDR ≤ 4.0 mm
Sella	1.14	1.45	74.0%	82.0%	94.0%
Nasion	1.29	1.37	64.0%	78.0%	94.0%
Orbitale	1.39	1.52	62.0%	76.0%	96.0%
Porion	1.39	1.68	68.0%	76.0%	88.0%
Point A	1.66	1.47	44.0%	70.0%	88.0%
Point B	1.24	1.61	68.0%	82.0%	94.0%
Pogonion	1.11	1.04	58.0%	86.0%	98.0%
Menton	1.21	1.10	62.0%	80.0%	98.0%
Gnathion	1.12	0.77	62.0%	84.0%	100.0%
Gonion	1.06	1.10	66.0%	86.0%	98.0%
md1-Inf	1.04	1.27	72.0%	88.0%	96.0%
mx1-Sup	1.09	1.55	76.0%	90.0%	94.0%
PNS	1.32	1.49	62.0%	76.0%	92.0%
ANS	1.36	1.61	62.0%	84.0%	94.0%
Articulare	1.29	1.50	68.0%	84.0%	92.0%
Overall	1.25	1.39	64.5%	81.5%	94.4%

Mean errors are reported in millimeters. The success detection rate (SDR) was calculated as the percentage of predicted landmarks with errors within predefined thresholds of 1.0, 2.0, and 4.0 mm. ANS, anterior nasal spine; md1-Inf, mandibular central incisor inferius; mx1-Sup, maxillary central incisor superius; PNS, posterior nasal spine; SD, standard deviation; SDR, success detection rate.

## 4. Discussion

The overall mean error was 1.25 ± 1.39 mm, with 81.5% of landmark predictions falling within the frequently used 2.0 mm threshold reported in previous studies [30]. These findings are encouraging. However, this threshold should be interpreted as a benchmark for model evaluation rather than evidence that all individual predictions are clinically acceptable. Because 18.5% of the predicted landmarks exceeded 2.0 mm, clinician review remains necessary before the estimated coordinates are used for diagnosis or treatment planning.

The holdout test set was independent of the training set, thereby providing an internal assessment of model performance on data not used for model fitting. The observed accuracy indicates favorable performance within this single-center test set. However, a single internal holdout design does not establish external generalizability or, by itself, demonstrate the absence of overfitting. In addition, all images were obtained within a single institutional imaging environment, and the model was not evaluated on images acquired using different radiographic systems, manufacturers, sensors, or exposure protocols. Therefore, these findings should be interpreted as internal validation only, and generalizability to other institutions, populations, imaging systems, and clinical workflows remains to be established [31].

Cephalometric landmark identification has been reported to depend on examiner experience and skill [22,32]. In this study, each cephalogram was annotated by one of five orthodontists, and the coordinates recorded for each image by the assigned orthodontist were used as the reference standard. Because none of the five orthodontists independently annotated the same images, interexaminer reliability could not be estimated using a fully crossed design [33]. To assess reference–annotation reproducibility, one orthodontist independently re-annotated all 50 test cephalograms after an interval of more than two weeks. The overall difference between the original and repeated annotations was 0.52 ± 0.92 mm. Although this value was smaller than the AI localization error of 1.25 ± 1.39 mm, both comparisons were referenced to the same original annotation; therefore, this finding should not be interpreted as evidence that the model performed within the range of interexaminer variability. Model performance was evaluated using Euclidean-distance errors and success detection rates, and the absence of a fully crossed interexaminer design remained a limitation.

Here, the SDR within 2.0 mm was 81.5%, compared with 74.8% reported by Wang et al. and 84.7% reported by Lindner et al. [5,30]. Because the datasets, landmark definitions, preprocessing procedures, and evaluation conditions differed across studies, these numerical values should not be interpreted as evidence that this model outperformed previously reported approaches. A single-stage model estimates landmark coordinates directly, without sequential coarse-to-fine stages, and may theoretically reduce opportunities for error propagation between stages. However, because no direct comparison with a multistage model was performed, this study does not demonstrate an architectural advantage.

Although the overall comparison among the 15 landmarks was statistically significant, the effect size was small, and descriptive, landmark-specific analyses showed relatively large mean errors for several landmarks in the cranial and maxillary regions. These landmarks share anatomical characteristics, including structural overlap and low contrast at the skull base, the bilateral external auditory meatus, and the ear rod [29,34]. Specifically, Orbitale and Porion are bilateral cranial landmarks; therefore, errors may occur even during manual measurement when determining the midpoint between the left and right structures [32]. This tendency was also observed in this study. Furthermore, because Point A is defined on a curved surface, substantial variation in its anteroposterior positioning has been reported [22]. Maxillary landmarks, including PNS and ANS, were similarly difficult to identify because of overlapping anatomical structures and reduced image contrast, which may have increased error [35]. In contrast, high accuracy was achieved for md1-Incisor inferius and Gnathion, which are located in the mandibular midline. These landmarks lie on the midsagittal plane and are therefore less susceptible to bilateral structural overlap, which may have contributed to their relatively high localization accuracy [36].

From a clinical perspective, the relatively larger errors at Porion and Point A require particular attention, as these landmarks are used in widely applied cephalometric measurements. Porion contributes to the construction of the Frankfort horizontal plane. Consequently, localization errors at this landmark may influence angular measurements based on this plane, such as the Frankfort mandibular plane angle. Point A is directly involved in sagittal skeletal assessments, including SNA and ANB, and errors at this landmark may affect the interpretation of maxillary position and skeletal classification. Therefore, when AI-generated coordinates are used clinically, orthodontists should carefully verify these anatomically ambiguous landmarks before diagnosis or treatment planning.

Herein, we adopted a single-stage regression CNN based on ResNet50. ResNet50 is a deep CNN with residual connections that supports feature extraction while mitigating vanishing gradients in deep network architectures [37]. The model directly regresses landmark coordinates from features extracted from lateral cephalograms via a single prediction pathway. More broadly, deep-learning approaches have been used to extract latent morphological features from mandibular image data [38]. However, this study did not directly evaluate the learned feature space or its contribution to landmark-localization performance. Although the architecture is conceptually straightforward, its relative advantages in accuracy, robustness, computational efficiency, or error propagation were not evaluated against a multistage model in this study. The training set included patients with a range of skeletal malocclusions; however, the extent to which this contributed to performance across morphological variation cannot be determined from the current design. No statistically significant differences were detected by sex or age group (*p* = 0.64 and *p* = 0.87, respectively), but the limited and unequal subgroup sizes preclude conclusions regarding equivalent performance across demographic groups.

AI-based automatic landmark detection has been reported to shorten measurement time compared with conventional manual methods [39]. However, because some predictions exceeded the 2.0 mm threshold and accuracy differed among landmarks and patients, the automatically generated coordinates should be reviewed and corrected by an orthodontist before being used for cephalometric analysis, diagnosis, or treatment planning. This model should therefore be regarded as a clinician-supervised support tool rather than an autonomous diagnostic system.

This study has some limitations. (1) The dataset included only lateral cephalograms from Japanese individuals, reflecting a specific ethnic background [21]. (2) The analysis was restricted to hard-tissue landmarks. (3) Although the overall mean error was below 2.0 mm, 18.5% of all landmark predictions exceeded the 2.0-mm threshold, and the success detection rate varied by landmark, including 70.0% for Point A; therefore, automated predictions require clinician verification. (4) In the reference–annotation reproducibility analysis, the original annotations were compared with remeasurements performed by a single examiner; as the five orthodontists did not independently annotate the same images, the true interexaminer variability among them was not assessed. (5) Repeated training with different random seeds was not performed; therefore, variability in model performance across independent training runs was not assessed. When this model is applied to other populations, differences in craniofacial morphology and skeletal characteristics may affect landmark-estimation accuracy. Furthermore, this study did not specifically evaluate model performance in patients with craniofacial anomalies, marked facial asymmetry, or orthodontic appliances. These conditions may alter landmark visibility, increase anatomical superimposition, or introduce radiopaque artifacts, thereby affecting automatic landmark detection. Future studies should include such clinically challenging cases to evaluate the robustness of the model across a broader range of orthodontic patients.

Statistical outliers were identified in 10 of the 50 test cases. Case No. 2 (SNA 89.8°, ANB + 9.4°) showed marked maxillary protrusion, whereas Case No. 49 (FMA 5.8°) showed an extremely hypodivergent skeletal pattern. However, a descriptive review of all 10 outlier cases did not identify a consistent pattern attributable to a specific landmark or skeletal morphology. Some cases showed uncommon craniofacial characteristics, whereas others showed relatively large errors at landmarks in regions of low image contrast, along curved anatomical contours, or in areas of bilateral structural overlap. Statistical outlier status does not necessarily indicate clinical failure, but the presence of these cases indicates that model performance was not uniform across patients. As this was an exploratory post hoc analysis involving a small number of cases, no statistical or causal association between specific patient characteristics and prediction error can be inferred.

In the single-center internal holdout test set, the model achieved a mean localization error of 1.25 mm. These findings support further evaluation of the approach as a clinician-supervised landmark-identification tool. Before broader clinical use, external validation and examiner-reliability studies using a fully crossed design, in which multiple orthodontists independently annotate the same images, are required [31,33,39].

## 5. Conclusions

Within this single-center internal holdout test set, a ResNet50-based single-stage regression CNN achieved a mean localization error of 1.25 ± 1.39 mm, with 81.5% of predictions within 2.0 mm. These findings indicate favorable internal test performance but do not establish superiority over multistage architectures, external generalizability, or decreased examiner dependence. As some predictions exceeded 2.0 mm, AI-generated coordinates should be regarded as outputs requiring orthodontist verification rather than as autonomous diagnostic results.

Nonetheless, this study used data from a single institution in Japan, and external validity remains unconfirmed. Furthermore, this study did not specifically evaluate model performance in patients with craniofacial anomalies, marked facial asymmetry, or orthodontic appliances. These conditions may alter landmark visibility, increase anatomic superimposition, or introduce radiopaque artifacts, thereby affecting automatic landmark detection. Future studies should include clinically challenging cases and external datasets from different institutions, populations, and imaging systems. Direct comparisons between single-stage and multistage architectures using the same dataset and evaluation protocol are also needed to determine whether the single-stage design provides any practical or performance advantage. Further studies should examine whether incorporating soft-tissue landmarks and additional model training improves landmark localization accuracy.

## Figures and Tables

**Table 1:** Definitions of the cephalometric landmarks.

Point No.	Landmark Name	Abbreviation	Landmark Description
Point 0	Sella	S	Center of the sella turcica
Point 1	Nasion	N	Most anterior point of the frontonasal suture
Point 2	Orbitale	Or	Most inferior point of the orbital margin
Point 3	Porion	Po	Most superior point of the external auditory meatus
Point 4	Point A	A	Deepest point on the anterior maxillary surface between the anterior nasal spine and the maxillary incisor alveolus
Point 5	Point B	B	Deepest point on the anterior surface of the mandibular symphysis between infradentale and pogonion
Point 6	Pogonion	Pog	Most anterior point on the mid-sagittal section of the mandibular mentum
Point 7	Menton	Me	Most inferior point on the mid-sagittal section of the mandibular mentum
Point 8	Gnathion	Gn	Constructed midpoint between Pogonion and Menton
Point 9	Gonion	Go	Constructed point at the mandibular angle that bisects the angle formed by the mandibular plane and ramus
Point 10	md1-Incisor inferius	Li	Incisal edge of the most anterior mandibular central incisor
Point 11	mx1-Incisor superius	Ls	Incisal edge of the most anterior maxillary central incisor
Point 12	PNS	PNS	Posterior nasal spine; the most posterior point of the hard palate
Point 13	ANS	ANS	Anterior nasal spine; the most anterior point of the nasal floor
Point 14	Articulare	Ar	Intersection of the posterior border of the mandibular ramus and the inferior surface of the cranial base

## Data Availability

The data presented in this study are available from the corresponding author upon request.

## Abbreviations

The following abbreviations are used in this manuscript:

AI	Artificial Intelligence
ANB	Point A–Nasion–Point B angle
ANS	Anterior nasal spine
CNN	Convolutional neural network
DICOM	Digital Imaging and Communications in Medicine
FMA	Frankfort mandibular plane angle
ICC	Intraclass correlation coefficient
Li	Incisor inferius
PNS	Posterior nasal spine
SDR	Success detection rate
